# The impact of a personalized, community-based counselling and referral programme on modern contraceptive use in urban Ghana: a retrospective evaluation

**DOI:** 10.1093/heapol/czaa082

**Published:** 2020-10-23

**Authors:** Elizabeth G Henry, Kristy M Hackett, Ayaga Bawah, Patrick O Asuming, Caesar Agula, David Canning, Iqbal Shah

**Affiliations:** 1 Department of Global Health and Population, Harvard T.H. Chan School of Public Health, Boston, MA, USA; 2 Regional Institute for Population Studies, University of Ghana, Legon, Accra, Ghana; 3 Department of Finance, University of Ghana Business School, University of Ghana, Legon, Accra, Ghana

**Keywords:** Family planning, contraception, Ghana, community health, impact, evaluation, health education, reproductive health, urban health

## Abstract

Community-based demand-generation family planning programmes have been associated with increased contraceptive use in rural areas of Ghana. However, rigorous evaluations of such programmes in urban contexts are lacking. We used a retrospective, cross-sectional with comparison group design to estimate the immediate and sustained impact of the Willows intervention on modern contraceptive use in Kumasi, Ghana. The Willows intervention is a home-based counselling and referral programme for women in low-income urban settlements. We analysed data from a cross-sectional representative survey of 1205 women of reproductive age in the intervention area and 1108 women in a matched comparison site. The main outcome was women’s reported contraceptive use at: (1) baseline (January 2013); (2) programme close (December 2016); and (3) follow-up (August to October 2018). We estimated the programme effect at the community level and for women who reported receiving a family planning counselling visit. We used coarsened exact matching to assess the impact of the intervention relative to outcomes for matched comparison women. Comparing those who reported a family planning visit in the intervention area with matched comparison area women who reported no visit, we estimated a 10.5 percentage point increase in use of modern contraceptives from baseline to close (95%CI : 6.2, 14.8; *P* < 0.001) and a 7.6 percentage point increase from baseline to follow-up (95%CI : 3.3, 11.9; *P* < 0.001). However, only 20.2% of women in the Willows intervention area reported a visit. The Willows intervention, therefore, did not achieve its aim to reach all reproductive-aged women in the community. At the community level, we found no significant effect of the intervention at either programme close or 2 years later. We recommend that similar community-based interventions strive for greater outreach and simultaneously launch robust prospective impact evaluations.

**Key Messages**

- Community-based family planning information, education and counselling programmes, usually combined with provision of family planning supplies and/or referrals, have been demonstrated to increase modern contraceptive use, but rigorous evaluations in urban African contexts are limited.
- Our study evaluated a 3-year personalized family planning counselling and referral programme in Kumasi, Ghana. We utilized a retrospective cross-sectional design with statistical matching to assess the impact of the intervention relative to outcomes for matched comparison women.
- Effect estimates from modelling with our matched sample indicate that the programme had a significant impact on modern contraceptive use at the close of the programme among women who received an information or counselling visit. This effect was sustained 2 years after the programme close; however, we found no significant effect at the community level due to low intervention coverage.
- While the home counselling and referral model holds promise in this setting, programmatic efforts to improve community coverage will be critical to maximizing impact on family planning outcomes.

## Introduction

Family planning is a proven intervention to prevent unintended pregnancies, related unsafe abortion, and maternal mortality and morbidity (Ahmed *et al.*, 2012). Modern contraceptives are the most effective family planning methods (Winner *et al.*, 2012). In Sub-Saharan Africa (SSA), there is a disconnect between women’s expressed fertility intentions, as measured by unmet need for family planning, and their use of modern contraceptives, which is largely attributable to fears of side effects and other health concerns (Van Lith *et al.*, 2013). This gap impedes progress on improving reproductive health outcomes, which are critical to achieving many of the Sustainable Development Goals (SDGs) (Starbird *et al.*, 2016). Improving access to and use of modern contraceptive methods is, therefore, central to achieving better health outcomes and SDGs.

Ghana has achieved important gains in family planning, including nearly universal awareness of any method of contraception and a nine percentage point increase in modern contraceptive prevalence rate (mCPR) nationally between 1998 and 2014 [Ghana Statistical Service (GSS) *et al.*, 2018]. However, despite significant investments in national family planning and reproductive health programmes and policies, there is still high unmet need for family planning, estimated at 30% among currently married women (GSS *et al.*, 2015). Moreover, even though it is one of the lowest in the region, the pregnancy-related mortality ratio has remained relatively static over the past decade (GSS *et al*, 2018).

The government of Ghana has committed to increasing the number of women and girls using modern contraception from 1.5 to 1.9 million through improved access to, and availability of, quality services, expanded contraceptive method mix and increased demand (Government of Ghana, 2017). However, the latest national mCPR of 25% (GSS *et al.*, 2018) is lower than the FP2020 target of 30% in the year 2020. In order to meet this target, programmes must also focus on both geographic areas and key populations in Ghana where modern method use is persistently low, including women living in urban areas (Beson *et al.*, 2018). Challenges specific to the Ghana context, including fear of side effects, concerns about menstrual irregularity, perceived safety risks and lack of effectiveness, attitudinal factors, and a preference, especially among young males and more highly educated females, for traditional methods (Aryeetey *et al.*, 2011; Hindin *et al.*, 2014; Machiyama and Cleland, 2014; Staveteig, 2016; Marston *et al.*, 2017), warrant a personalized approach. 

Studies indicate that community-based family planning programmes can generate demand by providing tailored information, education and counselling (IEC) on modern contraceptive methods, addressing concerns about side effects, and connecting women to existing services (Ezeh *et al.*, 2010). A recent review found that community-based IEC programmes, usually combined with provision of family planning supplies and/or referrals, can increase modern contraceptive use (Belaid *et al.*, 2016). In Ghana, the Navrongo Community Health and Family Planning Programme demonstrated that a combined nurse outreach/counselling and community-awareness approach increased modern contraceptive use (Debpuur *et al.*, 2002). But this programme was implemented in the rural Upper East region. There is some evidence to suggest that outreach by community health or family planning workers, in addition to exposure to local radio programmes, increases the use of modern methods in urban settings in Kenya, Nigeria and Senegal (as well as in India), (Speizer *et al.*, 2014) but questions remain about the effectiveness of this approach in poor, urban settings of Ghana.

### The Willows reproductive health programme

Since its launch in 1999 in Turkey, Willows International (‘Willows’) has implemented a community-based reproductive health behaviour-change model to improve access to and uptake of modern contraceptive methods. The goal of Willows is to empower women by increasing their awareness of their own health and reproductive rights, counselling and supporting them to adopt new reproductive health behaviors and referring them to existing services within their communities. Willows later expanded and has programmes operating in urban centres in Pakistan, Tanzania and Ghana. Willows has implemented in Ghana as part of the Reducing Maternal Mortality and Morbidity (R3M) initiative, a partnership between the government and five organizations that aimed to improve access to family planning and safe abortion services (Sundaram *et al.*, 2015).

Since 2013, R3M activities were implemented in the city of Kumasi, the second-largest city in Ghana located in the Ashanti region. In Kumasi, R3M included several activities to improve supply-side factors such as the expansion of operating hours of facilities to include nights and weekends in 2014 in order to provide family planning at more convenient times, and in 2016 the introduction of FP+, a programme that heavily subsidized the cost of long-acting reversible contraceptives in select government facilities. As part of this collaborative effort, Willows implemented a unique home-visit counselling and referral programme, which operated exclusively in two sub-areas of Kumasi. Following is a description of the Willows model as best we could ascertain through a review of programme documents, discussion with staff, and observation of current programme operations.

To implement the Willows model, the administrative team identifies an area for intervention in consultation with the local health and other government authorities, with a target of 20 000–50 000 women of reproductive age. These communities typically contain women of low socioeconomic status and have low mCPR despite a high density of facilities offering family planning services. The Willows model then aims to register all women of reproductive age, creating a community-wide database of women and their reproductive health needs and fertility intentions. This database is then used to stratify women into risk categories for unintended pregnancy, in order to prioritize women at greatest need for services and to provide tailored, individualized family planning counselling.

To achieve this, Willows trains and deploys field educators (FEs), a paid cadre of 25–35 men and women recruited from the local communities, to carry out home visits. During these visits, FEs inform women about the benefits of family planning, provide information on the range of available contraceptive methods and refer women to locally based healthcare services, when needed. FEs operate continuously in one designated sub-area, with each FE serving ∼700–1000 clients. The estimated average number of visits per client is about 4–5 over the course of the project, but it varies based on women’s reproductive statuses.

The database, also known as the Management Information System (MIS), captures and tracks a large number of variables collected during the registration period as well as during the implementation phase of the intervention. Using the registration data, women are classified initially into specific priority categories. Over the course of the programme’s implementation, changes in women’s status are tracked and they are re-classified when needed. The Willows model applies an algorithm that determines which women are the most in need of family planning service and, therefore, should be visited by the FE for education, information and referral (see Supplementary file S1). The MIS generates weekly lists of 60–70 priority women to receive home visits based on the priority setting algorithm. Women may be visited for a counselling visit or, for women who are new method adopters or have been referred to health facilities, a quick follow-up visit. Women not using any method of contraception with high risk of unintended pregnancy, ineffective users of traditional methods and unsatisfied users of modern methods are given high priority for home visits to provide education, information and referral service. Ineffective traditional users and women who are not using any method but do not want to have another child or have the next child after 2 years are scheduled to be visited by an FE once every 4–6 weeks. Women who are effective users of traditional methods, have used modern methods in the past 6 months and are satisfied, and those who wish to have a child and have given birth more than 2 years ago are visited less frequently (ideally once every 6 months).

In Kumasi, the Willows programme registered a total of 31 210 women during their initial registration period. Among these, nearly three-quarters were determined to be ‘at risk’ of pregnancy: women currently not using a method of contraception who did not want to have children in the next 2 years. Despite the protocol to enrol additional women who may have migrated to the area, just 2140 registrations were recorded after programme launch in late April 2013 and before January 2017 when the programme had ended.

Athough the Willows programme has expanded to serve over a million women in over 60 sites across Ghana, Pakistan, Turkey and Tanzania, to date, there have been no rigorous, independent evaluations conducted. The one unpublished external evaluation of the Willows model as implemented in six provinces of Turkey between 2004 and 2005 concluded that 65% of women served by Willows were using modern methods at the end of the programme, but did not include a comparison group design, instead comparing their findings with a nationwide mCPR estimate of 43% (Bulut *et al.*, 2006). Our study aimed to generate estimates of the effect of the Willows home-based counselling model as implemented in Kumasi, Ghana from 2013 to 2016 in order to guide future programming for community-based family planning behaviour-change interventions in urban Ghana and similar West African settings.

## Materials and methods

**Figure 1:**
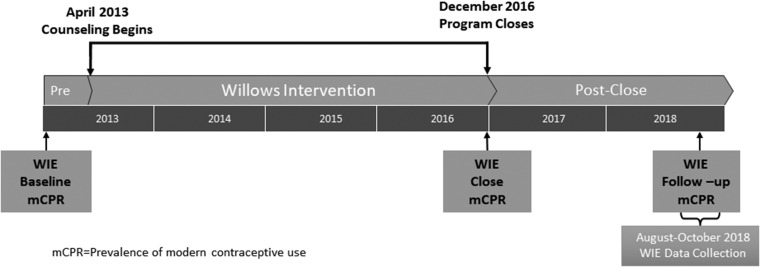
Willows impact evaluation (WIE) timeline.

Using a cross-sectional survey, we retrospectively assessed changes in women’s contraceptive use over the 5 years prior to the survey, including before the Willows programme launch, immediately following the end of the programme, and ∼18 months after the close of the programme. We conducted a household survey with ∼2200 women of reproductive age (16–49 years) in the intervention site where the programme ended 18 months prior, plus an additional 2200 women in a matched comparison site. The survey collected retrospective data on monthly contraceptive use from January 2013 (4 months before the Willows programme launch) to the month of data collection, which we conducted from August to October 2018 in both intervention and matched comparison areas within Kumasi Metro. We compared the rates of modern contraceptive use in intervention and comparison sites to estimate both immediate and sustained programme effect among a panel sample of women who had lived in the intervention and comparison areas continuously from the time of the programme’s launch in early 2013. Figure 1 illustrates the Willows Impact Evaluation (WIE) timeline, including the timing of programme implementation, data collection and primary outcome (mCPR) assessment.

### Study setting

The intervention site covered two neighbourhoods of Kumasi Metro: Aboabo and Sepe Buokrom and was selected by Willows in collaboration with the Ghana Health Service for the home-visit programme in order to reach women in poorer areas of the city with historically low use of contraceptive methods. Both neighbourhoods are densely populated and are comprised of a low-income population with migrants from various regions in Ghana. Aboabo is predominantly a Muslim community, located around the central mosque, whereas Sepe Buokrom, which is slightly less densely populated, is more religiously diverse. We purposively selected two additional communities to serve as comparison areas for the study: Sepe Timpon and Anloga, based on similarities in demographics, ethnicity and socioeconomic characteristics. Both Sepe Timpon and Anloga are considered to be predominantly poor and have higher rates of Muslim population than other areas of the city. In addition, there had not been any prior implementation of Willows in the comparison areas, and while they are neighbouring areas, they were not contiguous to the intervention sites, which minimized the potential for spillover effects. A map illustrating the locations of the intervention and comparison areas in Kumasi is included in the Supplementary material (S2).

### Sample size and sampling procedures

We powered our sample size to test for a statistically significant effect at 0.05 critical value with 90% probability of exceeding the critical value if the actual effect size in the intervention area would be at least a 5 percentage point difference in mCPR relative to the comparison group on a two-sided test. Our sample size calculation was pre-determined for our larger multi-country study that also included Willows interventions sites in Pakistan and Turkey. We assumed, on the basis of MIS programme data analysis of five closed sites across the three countries, a baseline CPR of 30% in each of the treatment and comparison areas with this baseline level continuing over the course of the project in the comparison area. This implied a required sample size of 1836 women in each of the intervention and comparison areas, which we rounded up to a sample of 2000 women in each site. We believed it advantageous to have the same sample size across sites, even with varying estimates of mCPR. In the case of Ghana, with a baseline mCPR estimate of 5.5% from the MIS data, the sample size needed to detect a 5 percentage point difference was estimated at 692, allowing us to limit our analyses to a panel of women who were resident in the study areas during the Willows registration period.

We used a multi-stage cluster random sampling approach to create a representative sample of women in each of the intervention and comparison sites. We constructed an original sampling frame for both areas. The research team worked closely with the Willows Ghana programme team to distinguish the boundaries for both the intervention and comparison sites. We then used population estimates and enumeration area (EAs) maps from the Ghana 2010 census, updated with expected population growth to 2018, to divide EAs into roughly equal-sized study clusters of ∼60 households. This yielded 1069 total clusters from 368 EAs. In each site, for the first stage we randomly selected 100 clusters (200 in total) and conducted a complete listing of all households within each cluster to identify eligible women. The eligibility criteria for women at the listing stage were (1) living in the study area at the time of survey and (2) age 16–49 years at time of survey. At the second stage, we randomly selected 25 households with at least one eligible woman per cluster for interviews in order to reach the target of 22 women due to the recruitment eligibility criteria and estimated refusals. Two additional eligibility criteria were assessed during the recruitment process (1) ability to communicate in one of three local languages (Ewe, Twi or Hausa) and (2) no diminished or limited mental capacity. Finally, at the third stage, if a household had more than one eligible woman, we randomly selected one woman from the household prior to the interview using a predetermined, randomly ordered list.

For this study, we restricted our analysis sample to a panel of women who were between the ages of 16 and 44 in 2013 and had lived in the area continuously from January 2013 to the day of data collection, as they would have had the maximum likelihood of exposure to the intervention. Although it is possible that women who moved into the intervention area after the initial registration period were later enrolled and counselled, as stated previously, there were low rates of registration of in-migrant women in the years after programme launch. Moreover, the characteristics of women who in-migrated between 2013 and 2018 differ from our panel sample on nearly all background characteristics except level of education. A table illustrating this is included in the Supplementary material (S3). A higher proportion of in-migrant women were younger (20–35 years of age), never married, non-Muslim and had fewer children. They also had a higher mCPR in 2018 than our panel. Therefore, we chose to exclude women who moved in after programme launch in order to estimate the effect among women most likely to have been reached. Finally, since the programme was aiming to intervene with women of reproductive age, we limited our analyses to women who would have been 16 years or older in 2013.

From a listing of 8109 households with eligible women in both intervention and comparison areas, 4584 women were sampled for interview. We had a contact rate of 92.3%, with some women having moved or migrated between the listing and the survey or being unable to locate despite several attempts. Our final sample included 4230 women (2168 in the intervention site and 2062 in the comparison site) yielded a consent rate of 98.5% of those contacted. Our panel sample of women produced a total sample of 2313 (1205 in the intervention and 1108 in the comparison site).

### Data collection

We used an electronic tablet-based survey to collect data on household characteristics and assets, women’s demographic characteristics, reproductive history and contraceptive use. This included a contraceptive calendar, modified from the Demographic and Health Survey (DHS) to include emergency contraception and designed for use on the tablet. The calendar module posed a series of questions to record all births, pregnancies and terminations over a specified period, with additional probing about contraceptive use between events to generate a complete month-by-month record of contraceptive history. The calendar collected, retrospectively, nearly 6 years of contraceptive use data from pre-Willows baseline (January 2013) until the survey in 2018. This approach has been used to record histories of 5–6 years prior to survey by the DHS and is widely accepted as a valid method for reproductive events up to the last 4 years (Bradley *et al.*, 2015). However, recall errors can increase as one goes back five or more years.

### Measures

The primary outcome for our analysis was mCPR, calculated as the percentage of women using a modern contraceptive method among all women of reproductive age. We calculated mCPR by using self-reported use of a modern contraceptive method, expressed as a binary variable (not using or using a modern method) for the month of January 2013 (baseline), December 2016 (immediate effect at Willows close) and the day of the survey (sustained effect at follow-up). Consistent with recent Ghana Maternal Health Survey and DHS definitions, modern contraceptive methods included male or female sterilization, intrauterine device, implants, injectables, oral contraceptive pills, male or female condoms, lactational amenorrhoea method and emergency contraception.

For our community-level analysis of the total sample, all women living in the intervention area were considered exposed to the intervention and those living in the comparison area were unexposed, since the intervention intended to cover all women of reproductive age in the area, with high anticipated rates of exposure. The main measure of exposure was living in the Willows intervention area continuously from January 2013 to the day of the survey.

We then conducted a second set of analyses accounting for reported exposure to programme. For this, we used a woman’s self-report of having had any visitor (Willows or otherwise) any time in the past 5 years to discuss pregnancy prevention or termination of unintended pregnancy. We also collected data on potential moderating variables including characteristics of the woman in 2013: age, marital status, education, religion, ethnicity, parity and modern contraceptive use at baseline.

### Data analysis

We first estimated the effect of the Willows programme by assessing differences in average mCPR at two separate time points: between baseline and close (immediate effect) and then between the baseline and follow-up (sustained effect) using a difference-in-differences (DID) estimation. This methodology accounts for observable and non-time-varying unobservable between-group differences by subtracting out baseline variance in the main outcome of interest (Dimick and Ryan, 2014). Our analysis used sampling weights to account for our sampling design and also accounted for clustering and study arm stratification.

Given the non-experimental design of our evaluation, we next conducted a matched analysis to ensure balance between the two study groups at the two different time points. Matching allowed us to control for some of the potentially confounding influences of baseline characteristics by reducing the imbalance between our intervention and comparison groups. We chose to apply coarsened exact matching (CEM) techniques to create a comparison of each treated woman with a matched ‘control’ woman from the comparison area, following the approach of Blackwell *et al.* (2009). First, we selected among the following observable baseline (2013) characteristics that we believed were important mediators in the relationship between exposure and the outcome. We then temporarily coarsened the data, creating sub-categories or bins for each variable in which individual women would be grouped: age group (16–19, 20–24, 25–29, 30–34, 35–39, 40–44), marital status (ever married, never married), education level (none, primary, secondary or higher), ethnicity (Akan, Ewe, Hausa, Mole-Dagbani or other), religion (Christian/other or Muslim), parity (0–1 or 2+ children) and baseline use of modern contraceptives (yes or no). Next, we performed exact matching on the women’s coarsened, or binned, data. Finally, we ran a general linear regression model on the uncoarsened, matched data to estimate programme effects. Recent work using simulation data suggests that in finite samples an alternative matching approach, the Propensity Score Matching method reduces balance and increases bias, while CEM increases balance and reduces bias (King and Nielsen, 2019).

We used STATA 15 (StataCorp.) for all analyses.

## Results

Table 1 illustrates the characteristics of the full study sample at both baseline (January 2013) and at the follow-up (August to October 2018). At baseline, relative to the comparison area, women in the Willows intervention site were more likely to be married (62.2% compared with 56.9%, *P* < 0.05), have lower levels of education (36.4% with no education compared with 31.9%, *P* < 0.01), and more likely to be Muslim (63.0% compared with 22.5%, *P* < 0.01). The comparison group had women of predominantly Akan ethnicity (62.8%, compared with 33.4%) whereas the ethnic composition of the intervention group was more diverse. There was no difference in parity between women in the two sites. However, the comparison site had a higher rate of modern contraceptive use at baseline than the intervention site (9.3% compared with 6.5%, *P* < 0.05).

**Table 1:** Characteristics of the retrospective panel sample: women living in study areas and ages 16–44 in January 2013

	January 2013 (Baseline)		August to October 2018 (follow-up)	
	Intervention	Comparison	Intervention	Comparison
Characteristics	(*n* = 1205)	(*n* = 1108)	(*n* = 1205)	(*n* = 1108)
Age group (years)				
16–19	163 (13.5%)	143 (12.9%)		
20–24	266 (22.1%)	212 (19.1%)	163 (13.5%)	143 (12.9%)
25–29	213 (17.7%)	229 (20.7%)	266 (22.1%)	212 (19.1%)
30–34	256 (21.2%)	216 (19.5%)	213 (17.7%)	229 (20.7%)
35–39	167 (13.9%)	145 (13.1%)	256 (21.2%)	216 (19.5%)
40–44	140 (11.6%)	163 (14.7%)	167 (13.9%)	145 (13.1%)
45–49			140 (11.6%)	163 (14.7%)
Marital status				
Married/union	749 (62.2%)	631 (56.9%)*	743 (61.7%)	639 (57.7%)
Never married/union	388 (32.2%)	398 (35.9%)	337 (28.0%)	325 (29.3%)
Former Union	68 (5.6%)	79 (7.1%)	125 (10.4%)	144 (13.0%)
Education				
None	439 (36.4%)	354 (31.9%)**	410 (34.0%)	297 (26.8%)**
Primary	203 (16.8%)	119 (10.7%)	184 (15.3%)	118 (10.6%)
Middle/Junior Secondary	362 (30.0%)	396 (35.7%)	324 (26.9%)	406 (36.6%)
Secondary	146 (12.1%)	174 (15.7%)	205 (17.0%)	192 (17.3%)
Higher	43 (3.6%)	45 (4.1%)	77 (6.4%)	93 (8.4%)
Missing	12 (1.0%)	20 (1.9%)	5 (0.4%)	2 (0.1%)
Religion^a^				
Catholic			46 (3.8%)	64 (5.8%)**
Pentecostal			190 (15.8%)	510 (46.0%)
Other Christian			203 (16.8%)	261 (23.6%)
Muslim			759 (63.0%)	249 (22.5%)
Other/none			7 (0.6%)	24 (2.2%)
Ethnicity^a^				
Akan			403 (33.4%)	696 (62.8%)**
Ewe			11 (0.9%)	140 (12.6%)
Hausa			249 (20.7%)	47 (4.2%)
Mole-Dagbani			245 (20.3%)	71 (6.4%)
Other			297 (24.6%)	154 (13.9%)
Parity				
0	397 (32.9%)	357 (32.2%)	263 (21.8%)	223 (20.1%)
1	191 (15.9%)	186 (16.8%)	144 (12.0%)	154 (13.9%)
2	199 (16.5%)	190 (17.1%)	188 (15.6%)	184 (16.6%)
3	182 (15.1%)	179 (16.2%)	202 (16.8%)	191 (17.2%)
4+	234 (19.4%)	195 (17.6%)	403 (33.4%)	351 (31.7%)
Missing	2 (0.2%)	1 (0.1%)	5 (0.4%)	5 (0.5%)
Modern contraceptive use	78 (6.5%)	103 (9.3%)*	154 (12.8%)	181 (16.3%)*

Tests are between intervention and comparison groups at each time point

* *P* < 0.05;

** *P* < 0.01.

a Both assessed only for 2018 at time of the survey.

At the follow-up, there were some differences in the two groups relative to baseline, as would be expected as women age. More women had completed secondary school 5 years later in both groups, and in both groups women had more children. In both study area, nearly the same proportion of women were married at the follow-up (61.7% compared with 62.2% in the intervention and 57.7% compared with 56.9% in comparison) whereas fewer women had never been married in both groups, and more women reported formerly being in union or married.

**Figure 2:**
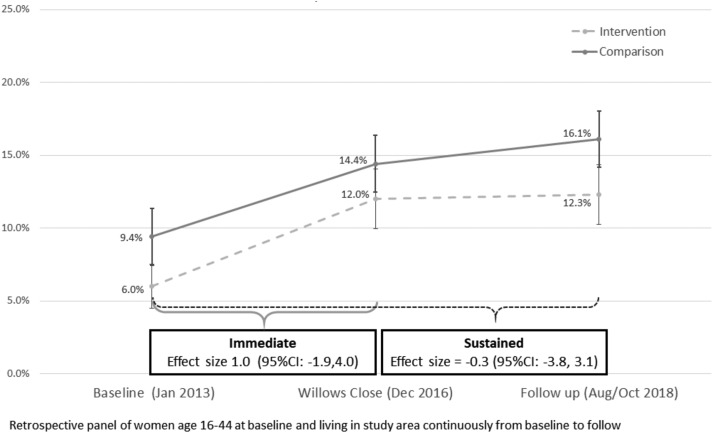
Modern contraceptive prevalence, intervention and comparison areas of Kumasi, Ghana.

Figure 2 illustrates the immediate and sustained DID estimates at the community level for our panel sample. Without adjusting for between-group differences, there was a non-significant immediate increase of 1.0 percentage point (95% CI: −1.9, 4.0, *P* = 0.50) and 0.3 percentage point decrease in mCPR between baseline and follow-up (95% CI: −3.8, 3.1, *P* = 0.85).

Adjusting for the baseline differences between intervention and comparison sites with our matched dataset, we found no significant effect on mCPR from baseline to either programme close or follow-up on the full sample (Table 2). CEM analysis matched 87% of intervention women and 93% of comparison women across 128 strata out of a possible 248, with an L1 statistic of 0.25, reduced from 0.52. A table comparing the demographics of the unmatched and matched full samples are included in the Supplementary materials (S4). We observed a 2.2 percentage point non-significant increase in intervention over comparison, from baseline to close (95%CI: −1.0, 5.4, *P* = 0.17) and an almost identical non-significant sustained effect of 2.2 percentage points from baseline to follow-up (95%CI: −1.0, 5.5, *P* = 0.18).

**Table 2:** Results of coarsened exact matching analysis, full sample and family planning visit sample

	Full sample		Family planning visit sample	
	Intervention	Comparison	Intervention with an FP visit	Comparison with no FP visit
Matched women^a^	1046	1027	218	781
Total women	1205	1108	244	1079
% Women matched	86.8%	92.7%	89.3%	72.4%
Matched strata	128		68	
Total strata	248		205	
% Strata matched	51.6%		33.2%	
L1 Statistic: Before	0.52		0.61	
After	0.25		0.25	
Effect size, mCPR:				
Baseline to close	2.2		10.5^**^	
	(95%CI: −1.0, 5.4)		(95%CI: 6.2, 14.8)	
Baseline to follow-up	2.2		7.5^**^	
	(95%CI: −1.1, 5.5)		(95%CI: 3.3, 11.8)	

a Matching variables included characteristics from 2013: marital status, education, age, religion, ethnicity, parity and baseline use of modern contraceptives.

* *P* < 0.05;

** *P* < 0.01.

In our sample of the intervention site, only 20.2% (*n* = 244/1205) of women reported receiving a family planning visitor in the past 5 years, compared with only 2.2% (*n* = 24/1108) of women in the comparison site, and this difference is statistically significant. While this suggests that women in the intervention area had a greater likelihood of having been visited by someone from the Willows programme, the level of coverage was lower than expected, and may have been insufficient to result in an observable community-level change in mCPR.

Table 3 describes the 2013 characteristics of the sub-sample of women who reported a visit, compared with those also in the intervention area who did not report a visit. A higher proportion of women who received a visit were between the ages of 25–39 years and were married. There was no significant difference between the groups with respect to education level, religion, ethnicity, parity or baseline modern contraceptive use.

**Table 3:** Characteristics of women living in intervention area and ages 16–44 in January 2013 by exposure to a family planning visit

January 2013 (baseline) characteristics	Report having had an FP visit	No reported visit
	(*n* = 244)	(*n* = 945)
Age group (years), *n* (%)		
16–19	25 (10.2)	138 (14.6)^**^
20–24	53 (21.7)	208 (22.0)
25–29	51 (20.9)	160 (16.9)
30–34	64 (26.2)	189 (20.0)
35–39	36 (14.8)	129 (13.7)
40–44	15 (6.1)	121 (12.8)
Marital status, *n* (%)		
Married/union	171 (70.1)	567 (60.0)^*^
Former union	8 (3.3)	59 (6.2)
Never married/union	65 (26.6)	319 (33.8)
Education, *n* (%)		
None	88 (36.1)	347 (36.7)
Primary	43 (17.6)	154 (16.3)
Middle/junior secondary	76 (31.1)	284 (30.1)
Secondary	26 (10.7)	119 (12.6)
Higher	10 (4.1)	34 (3.6)
Missing	1 (0.4)	7 (0.0)
Religion,^a^*n* (%)		
Catholic	6 (2.5)	39 (4.1)
Pentecostal	41 (16.8)	145 (15.3)
Other Christian	37 (15.2)	165 (17.5)
Muslim	159 (65.2)	590 (62.4)
Other/none	1 (0.4)	6 (0.6)
Ethnicity,^a^*n* (%)		
Akan	89 (36.5)	309 (32.7)
Ewe	5 (2.0)	6 (0.6)
Hausa	49 (20.1)	194 (20.5)
Mole-Dagbani	49 (20.1)	194 (20.5)
Other	52 (21.3)	242 (25.6)
Parity, *n* (%)		
0	69 (28.3)	325 (34.4)
1	39 (16.0)	149 (15.8)
2	40 (16.4)	155 (16.4)
3	45 (18.4)	135 (14.3)
4+	51 (20.9)	179 (18.9)
Missing	0 (0.2)	2 (0.2)
mCPR, *n* (%)	19 (7.8)	58 (6.1)

* *P *< 0.05;

** *P* < 0.01.

a Both assessed in 2018 at time of the survey.

**Figure 3:**
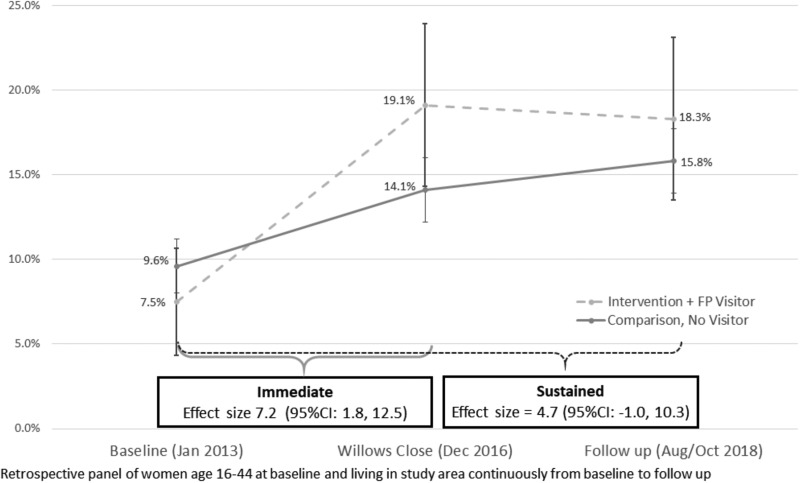
Modern contraceptive prevalence, intervention area women with a family planning visit and comparison area.

Figure 3 illustrates the DID estimates of mCPR at baseline, close and follow-up for women in the intervention area who reported a visit (*n* = 244) and women in comparison who reported none (*n* = 1079). Without adjusting for between-group differences, our difference-in-differences estimate was a 7.2 percentage point increase in mCPR (95%CI: 1.8, 12.5, *P* = 0.009) with a non-significant sustained estimate of 4.7 percentage points (95%CI: −1.0, 10.3, *P* = 0.11).

Results from the matched analysis, also shown in Table 2, reveal a more substantial, significant effect. CEM analysis matched 89% of intervention women with a visit and 72% of comparison women with no visit, across 68 strata out of a possible 205, with an L1 statistic of 0.25, reduced from 0.61. We observed a 10.5 percentage point increase in intervention over comparison, from baseline to close (95%CI: 6.2, 14.8, *P* < 0.001) and a 7.6 percentage point increase from baseline to follow-up (95%CI: 3.3, 11.8, *P* < 0.001). Table 2 summarizes results from the matched analysis for both community-level and family planning visit samples.

## Discussion

In this study, we sought to evaluate the intervention as implemented by Willows International in Kumasi, Ghana from 2013 to 2016. We observed a statistically significant immediate increase in modern contraceptive use as well as a significant sustained effect among the 20% of women who reported having a family planning visit since the time of the programme launch, relative to women in the comparison site who reported not being visited. However, this level of coverage was insufficient to result in an observable community-level change in mCPR.

National-level data indicate an overall positive trend in mCPR in Ghana, from 14.3% in September 2013 to 21.7% in September 2016—the highest annual rate of change among five SSA countries analysed (Burkina Faso, Ethiopia, Ghana, Kenya and Uganda) (Ahmed *et al.*, 2019). Regional data from Ashanti, in which Kumasi is located, show a similar trend (Kwame Nkrumah University of Science & Technology School of Medicine and The Bill & Melinda Gates Institute for Population and Reproductive Health at The Johns Hopkins Bloomberg School of Public Health, 2013, 2016). This underscores the importance of our study design, using a comparison group to account for other potential causes of any observed increases. Evaluations of urban-based family planning programmes have historically lacked in rigour due to the challenge of finding a suitable comparison group (Speizer *et al.*, 2014). Though we faced a similar challenge in our study, we were able to employ statistical matching techniques with CEM to create balance between the groups in order to assess the programme effect on the matched women.

There are several demand-generation family planning programmes that have demonstrated similar findings to our study. For example, the Navrongo project, which included both nurse outreach and community mobilization, as well as supplying contraceptives, showed a statically significant but small 2.2 percentage point increase in mCPR from 1993 to 1999, similar to what we observed at the community level (Debpuur *et al.*, 2002). A randomized trial conducted in Ethiopia in the early 1990s demonstrated strong evidence of an effect of home-based couples counselling on modern contraceptive use at 12 months, with twice as many couples using modern methods at 12 months in the experimental area relative to their control (Terefe and Larson, 1993). A recent cluster randomized trial of a programme where health surveillance assistants counselled young couples in Malawi on family planning led to an increase in method uptake 6 months post-intervention, though the results were not conclusive. A programme similar to Willows but conducted in a very different context in Jordan, with home-based couples counselling, showed no difference between intervention and control (1.5 percentage points). However, these programmes focused mostly in rural contexts, and not in urban centres.

As stated earlier, an important piece of contextual information to consider when interpreting our findings is that the Willows model only operated in the intervention area, and our evaluation is isolating the Willows model’s effect among those exposed to all R3M activities. It is likely that several of the supply-side activities implemented by R3M partners may have influenced the contraceptive use behaviors of women living in all of Kumasi Metro, including both the intervention and comparison areas. In addition, the government’s Reproductive Health Service Policy and Standards were revised in 2014 to allow community health nurses to provide implants, and the widespread promotion of Nexplanon (a new implant) to improve method mix in 2015 (GHS, 2016). Thus, the change in mCPR in our study should be interpreted as an increase above what would have been observed if only the other R3M and government activities had been implemented. It is possible that if the Willows model had been implemented in Kumasi in the absence of citywide R3M activities—as it is done in other countries—we might have observed a larger effect.

Few evaluations have assessed the impact of family planning demand-focused interventions, and none of the programmes evaluated focus solely on home-based counselling delivered over an extended period and examined sustained effects. Our study in a new urban setting that focuses on a behaviour-change, demand-generation model for improving contraceptive prevalence for nearly 2 years after a programme has ceased operations is unique and contributes to the evidence base for community-based demand-generation family planning programmes. Despite the intended aim to cover the entire community, our data indicate that Willows’ overall reach was low. A far higher proportion of women in Kumasi were at risk for unintended pregnancy at registration than for other countries with higher baseline mCPR. By following the same prioritization scheme and implementation procedures, including a similar weekly case load for FEs as in other countries, it is possible that the lower-priority women received few if any visits over the course of the programme’s implementation. Given that 25% of women are estimated to discontinue any method within 12 months (at a national level) (GSS *et al.*, 2015), women in this and other priority groups may be moved disproportionately in and out of users groups, contributing to lower overall mCPR at the community level. Our findings underscore the need for investment in process evaluation of the model in order to understand how the implementation in this context may have influenced the outcomes we observed and identify opportunities for improvements.

## Limitations

Our study has some limitations. We understand that Willows targeted areas of Kumasi that were hardest to reach and had the lowest estimates of mCPR. It is possible that the comparison area we selected did not serve as an appropriate counterfactual. The change observed in the comparison area in our study may have been greater than what would have been observed in the intervention area absent Willows. If this is the case, then we may have underestimated the programme effect. In addition, one key assumption of our analysis is that there are no unobserved covariates that correlated with treatment and affect the outcome of interest. We did our best to select an appropriate comparison group in the design phase, but it is possible that other policies or programmes were implemented differentially between the two sites. We did document other programme or policy changes that may have influenced the intervention or comparison sites differently in order to aid in our interpretation of the data. Moreover, our use of CEM mitigated the issues of imbalance between the groups especially in terms of religion, and we were able to match 87% of intervention women.

Another limitation of our retrospective approach is that we could not collect data contemporaneously; the survey relied on women’s self-report of past events and is therefore subject to recall error bias. However, our intervention mCPR estimate is nearly the same as the mCPR captured in the baseline registration for the Willows project, giving us more confidence that it reflects the population estimate from 2013.

## Conclusion

This study provides new information about the effect of a community-based demand-generation family planning counselling and referral programme in an urban setting in Ghana and contributes uniquely to the evidence base for the immediate and sustained effects of family planning programmes. While results indicate a promising increase in modern method use for women who received a family planning visit, we do not know the extent to which the Willows programme was the sole cause of this improvement. We identified an implementation effect of the programme in the intervention site, but the rate of exposure was much smaller than what the Willows intervention intended. This may be why we observed no effect of the programme on the community as a whole. We recommend a prospective, experimental or quasi-experimental evaluation of family planning demand-generation programmes to determine the impact of such initiatives concentrating on targeting vulnerable populations in densely populated, urban settings.

## Supplementary data

Supplementary data are available at *Health Policy and Planning* online.

## Supplementary Material

czaa082_Supplementary_Data
