# Supplementary figures and images for: The impact of a personalized, community-based counselling and referral programme on modern contraceptive use in urban Ghana: a retrospective evaluation
Source: Health Policy Plan. 2020 Oct 23;35(10):1290–9. doi: 10.1093/heapol/czaa082 (PMC7886439; doi:10.1093/heapol/czaa082)

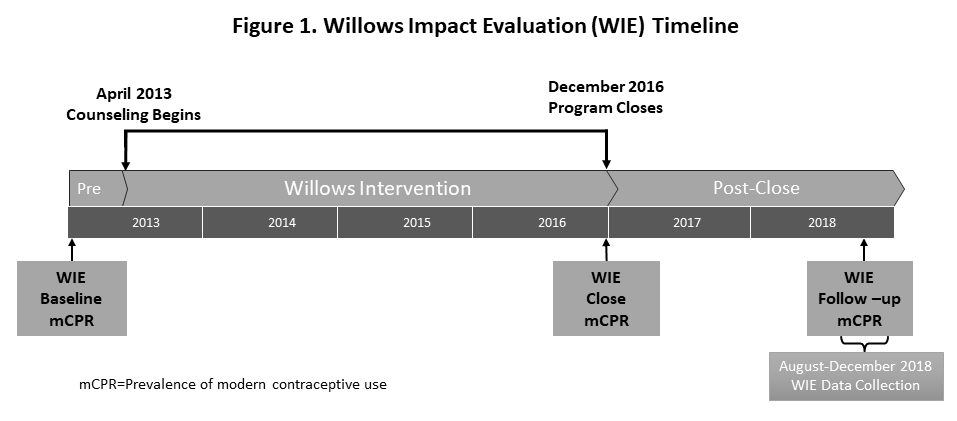

Supplement: czaa082_Supplementary_Data [file czaa082_supplementary_data.zip › Fig1_rev1.tif]

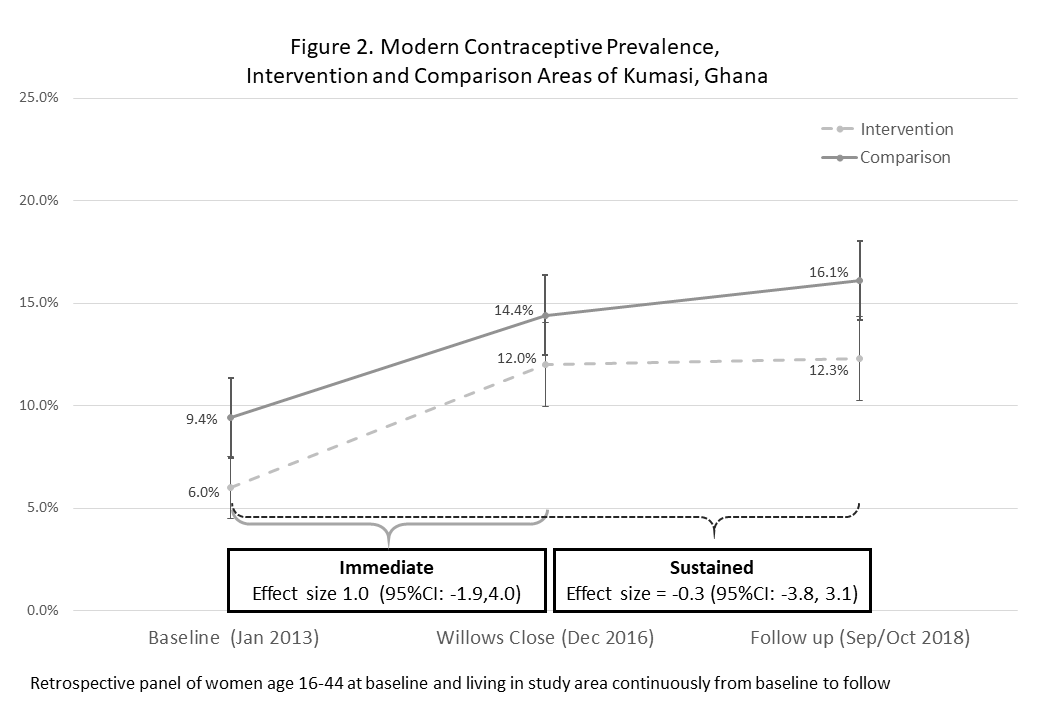

Supplement: czaa082_Supplementary_Data [file czaa082_supplementary_data.zip › Fig2_rev1.tif]

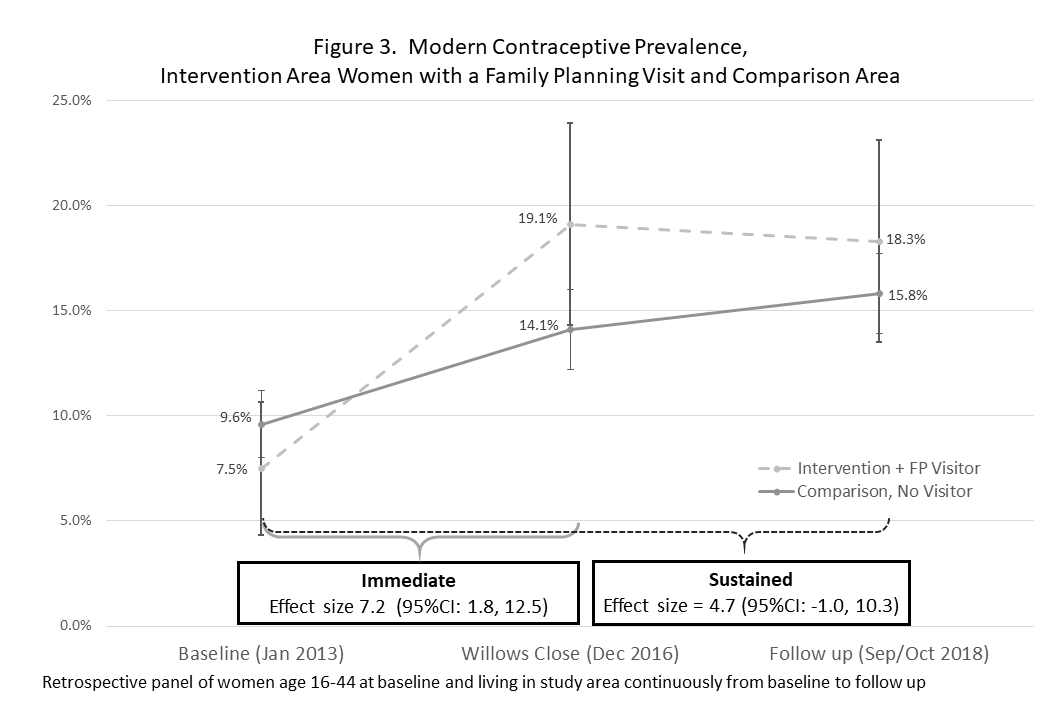

Supplement: czaa082_Supplementary_Data [file czaa082_supplementary_data.zip › Fig3_rev1.tif]

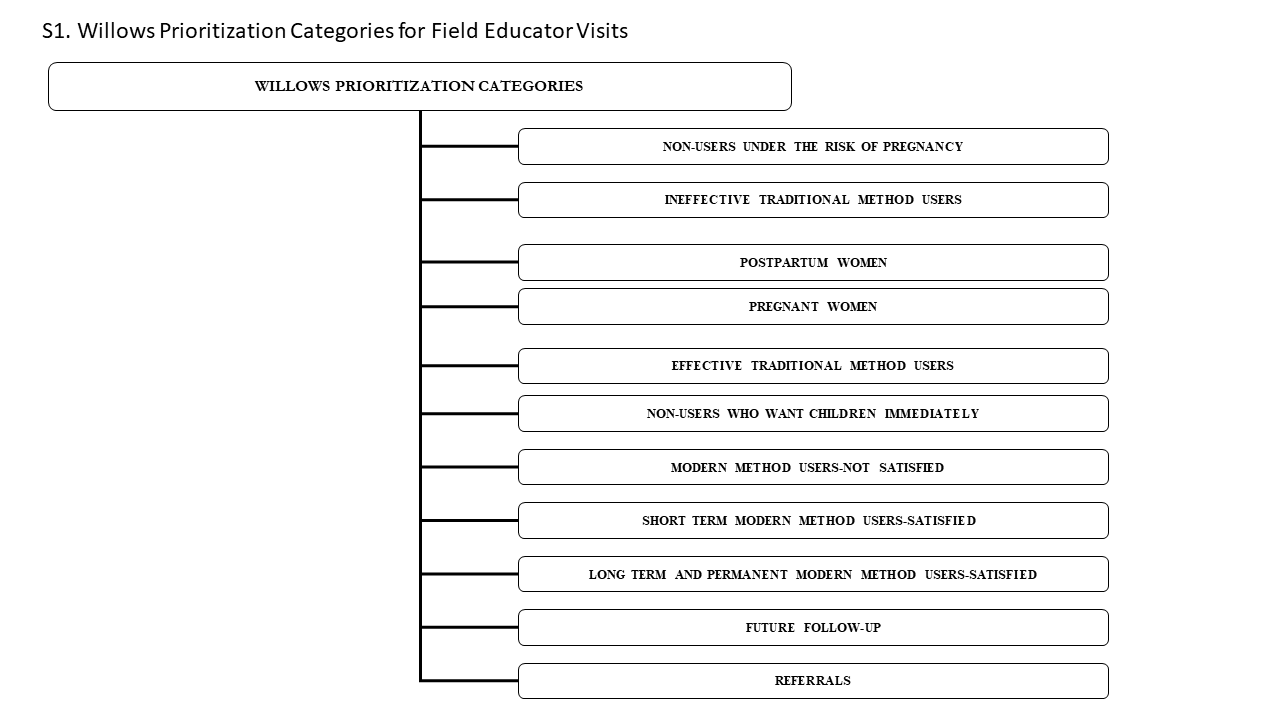

Supplement: czaa082_Supplementary_Data [file czaa082_supplementary_data.zip › S1. Algorithm.tif]

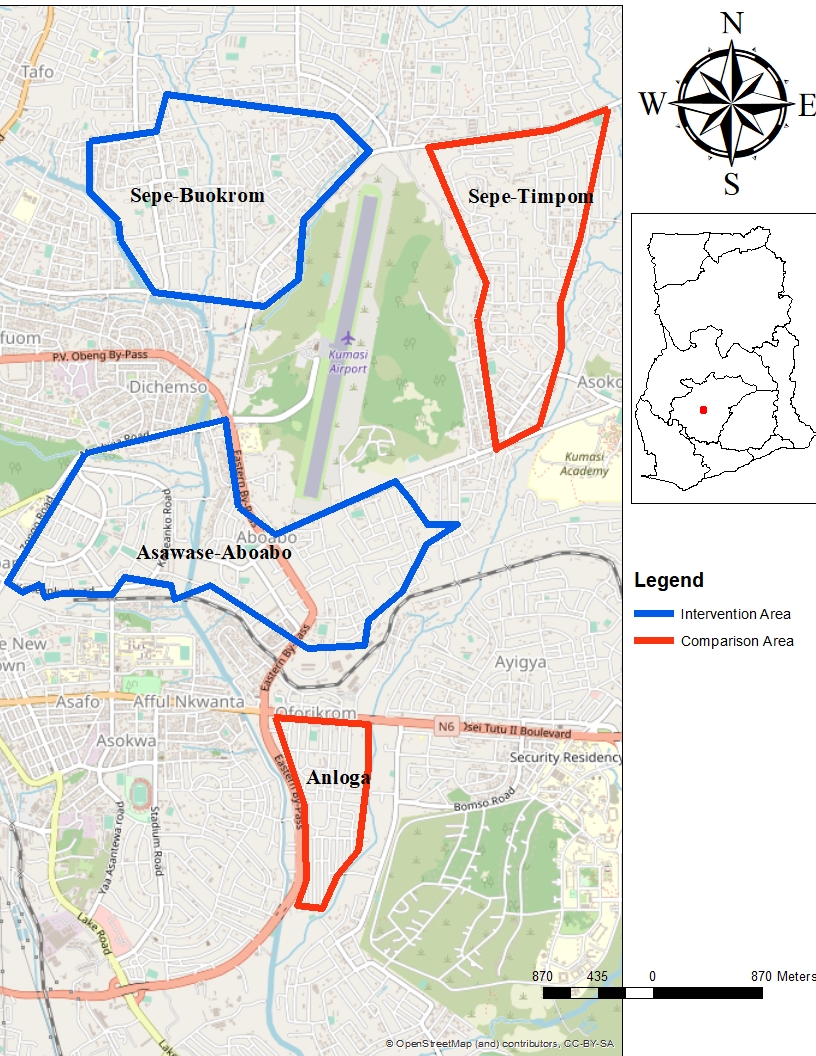

Supplement: czaa082_Supplementary_Data [file czaa082_supplementary_data.zip › S2. Kumasi Map.jpg]
